# Hexachloronaphthalene Induces Mitochondrial-Dependent Neurotoxicity via a Mechanism of Enhanced Production of Reactive Oxygen Species

**DOI:** 10.1155/2020/2479234

**Published:** 2020-06-26

**Authors:** Malwina Lisek, Joanna Stragierowicz, Feng Guo, Philipp P. Prosseda, Magdalena Wiktorska, Bozena Ferenc, Anna Kilanowicz, Ludmila Zylinska, Tomasz Boczek

**Affiliations:** ^1^Department of Molecular Neurochemistry, Medical University of Lodz, 92215 Lodz, Poland; ^2^Department of Toxicology, Medical University of Lodz, 90151 Lodz, Poland; ^3^Department of Pharmaceutical Toxicology, China Medical University, Shenyang 110122, China; ^4^Department of Patho Biochemistry, Goethe Universitätsklinikum, 60590 Frankfurt am Main, Germany; ^5^Department of Molecular Cell Mechanisms, Medical University of Lodz, 92215 Lodz, Poland

## Abstract

Hexachloronaphthalene (PCN67) is one of the most toxic among polychlorinated naphthalenes. Despite the known high bioaccumulation and persistence of PCN67 in the environment, it is still unclear to what extent exposure to these substances may interfere with normal neuronal physiology and lead to neurotoxicity. Therefore, the primary goal of this study was to assess the effect of PCN67 in neuronal *in vitro* models. Neuronal death was assessed upon PCN67 treatment using differentiated PC12 cells and primary hippocampal neurons. At 72 h postexposure, cell viability assays showed an IC_50_ value of 0.35 *μ*g/ml and dose-dependent damage of neurites and concomitant downregulation of neurofilaments L and M. Moreover, we found that younger primary neurons (DIV4) were much more sensitive to PCN67 toxicity than mature cultures (DIV14). Our comprehensive analysis indicated that the application of PCN67 at the IC_50_ concentration caused necrosis, which was reflected by an increase in LDH release, HMGB1 protein export to the cytosol, nuclear swelling, and loss of homeostatic control of energy balance. The blockage of mitochondrial calcium uniporter partially rescued the cell viability, loss of mitochondrial membrane potential (*ΔΨ*_m_), and the overproduction of reactive oxygen species, suggesting that the underlying mechanism of neurotoxicity involved mitochondrial calcium accumulation. Increased lipid peroxidation as a consequence of oxidative stress was additionally seen for 0.1 *μ*g/ml of PCN67, while this concentration did not affect *ΔΨ*_m_ and plasma membrane permeability. Our results show for the first time that neuronal mitochondria act as a target for PCN67 and indicate that exposure to this drug may result in neuron loss via mitochondrial-dependent mechanisms.

## 1. Introduction

Polychlorinated naphthalenes (PCNs) were included in 2015 into the Stockholm Convention on Persistent Organic Pollutants to protect human health and the environment from highly dangerous, long-lasting chemicals by the elimination of their production and reduction of their unintentional release [1]. Despite these restrictions and no commercial use for over 30 years, the general population is constantly exposed to PCNs due to their main accumulation in foods of animal origin, especially those rich in fat (oil from fish, meat, and milk) [2–5]. Currently, the main sources of PCNs are not old technical formulations used previously (e.g., Halowax) but high-temperature industrial processes that contribute to global environmental contamination [6–10]. The evidence of their ubiquity in the environment is their detection in sediments, soil, water, and air [11–18]. In addition, high lipophilicity and resistance to degradation have led to their bioconcentration and bioaccumulation in the food chain [19]. This is confirmed by their persistent presence in various biological materials collected from the general population such as serum [20, 21], liver [22], adipose tissue [23], human milk [24, 25], and umbilical cord blood [26].

PCNs are often labelled as dioxin-like compounds (DLCs) not only because their mechanism of action (through the aryl hydrocarbon receptor (AhR)) is similar to polychlorinated dibenzo-*p*-dioxins (PCDDs), polychlorinated dibenzo-*p*-furans (PCDFs), and polychlorinated biphenyls (PCBs) [27, 28] but also primarily because of the toxic effects observed in occupationally exposed humans and experimental animals [29, 30]. Primarily, chloracne and hepatotoxic effects have been demonstrated in workers exposed by inhalation and dermal route to PCNs [31, 32]. In animal studies, orally administered PCNs have also shown hepatotoxicity [33, 34] and additionally hematological disorders [35, 36], thyroid and sex hormone disturbances [37], and prenatal toxicity [38–41]. Some nonspecific symptoms such as weight loss due to reduced appetite (anorexia), headaches, vertigo, and insomnia [42] have also been reported suggesting a potential neurotoxic effect of PCNs and the mechanism of action similar to DLCs. The hypothesis that PCNs can also target the central nervous system (CNS) is further supported by several studies showing their affinity for sciatic nerve as well as a spectrum of anorectic and behavioral effects in response to *in vivo* drug administration [34, 43–45]. While the mechanism by which PCNs can modify the behavioral pattern of animals is not known, studies performed both *in vivo* and *in vitro* suggest the action through GABAergic and/or glutamatergic systems [37, 46].

It has been estimated that one of the most toxic among all 75 known PCNs are hexachloronaphthalenes, mainly congeners 1,2,3,4,6,7-hexachloronaphthalene (PCN66), 1,2,3,5,6,7-hexachloronaphthalene (PCN67), and 1,2,3,6,7,8-hexachloronaphthalene (PCN70) [47, 48]. Besides, pentachloronaphthalenes, PCN66/67, are considered to be characteristic for combustion processes [12, 49, 50] and are most frequently detected in food [4, 51]. Hexachloronaphthalenes are also characterized by their highest bioaccumulation (especially in the liver, adipose tissue, and milk during lactation) as revealed in both humans and experimental animals [25, 52].

Despite widely documented harmful and adverse effects of PCN67, it is still unknown whether it may disturb neuronal function and produce subsequent neurotoxicity. To investigate the role of this substance in neurological processes, we employed *in vitro* models of primary hippocampal neurons and differentiated PC12. Here, we demonstrate that PCN67 disrupts neuronal sprouting and the formation of neurites. Moreover, our studies show the induction of mitochondrial-related necrotic death in a dose- and time-dependent manner. Our report is the first study, showing that mitochondria may be a primary intracellular target for PCN67 in neuronal cells.

## 2. Materials and Methods

### 2.1. Reagents

All reagents, if not separately mentioned, were purchased from Sigma-Aldrich. The PC12 rat pheochromocytoma cell line was obtained from the American Type Culture Collection (ATCC). Maxima SYBR Green Master Mix, M-MLV Reverse Transcriptase, Trizol®, Alexa Fluor 488, Lipofectamine LTX reagent, B27, and Neurobasal were from Thermo Fisher Scientific. Protein Assay Kit was from Bio-Rad. Anti-GAPDH (Cat. No. sc-32233) and anti-histone H3 (Cat. No. sc-517576) were from Santa Cruz Biotechnology. HMGB1 antibody (Cat. No. 3935) was from Cell Signaling Technology. LDH Cytotoxicity Assay Kit was from Cayman Chemical. GcAMP3 calcium sensor was a gift from Loren Looger (Addgene plasmid #22692). Primers were synthesized in the Institute of Biochemistry and Biophysics (Poland).

### 2.2. The Compound

The mixture of hexachloronaphthalene congeners (94.14% purity) used in this study contained 81.17% of PCN67 as a dominant congener and additionally congeners 1,2,3,4,6,7-; 2,3,4,5,6,7-; and 1,2,4,5,6,7-hexachloronaphthalene comprising together up to 12.98% and 5.85% of 1,2,3,4,5,6,7-heptachloronaphtalene. The synthesis, characteristic, and purity were described previously [33, 36, 37, 40, 45]. The analysis using the HRGC/HRMS method showed that the content of PCDDs and PCDFs was below 0.1 pg/mg.

### 2.3. PC12 Cell Culture, Differentiation, and PCN67 Treatment

PC12 cells derived from pheochromocytoma were routinely grown in collagen-coated (type I from rat tail) plastic dishes in RPMI 1640 medium supplemented with 10% horse serum, 5% fetal bovine serum, 25 mM HEPES, pH 7.4, 2 mM L-glutamine, 1 mM sodium pyruvate, and the mix of penicillin/streptomycin in a humidified incubator at 37°C with 5% CO_2_. Cells were plated at the density of 1‐2 × 10^5^/ml and cultured for 2 days before PCN67 administration. PCN67 was dissolved in DMSO and was added together with 1 mM dibutyryl-cAMP (differentiating agent). Cells were cultured with both agents for up to 72 h. No more than 20 cell passages were used for all experiments. Cells simultaneously cultured in the presence of 0.1% DMSO were used as a control. The black/white pictures of cell morphology were taken using an Olympus CK-40 inverted microscope equipped with a CCD camera.

### 2.4. Quantification of PC12 Cell Differentiation

We used two parameters to score the potency of PC12 cell to differentiate into neuronal phenotype: the length of the longest neurite (any protrusion longer than the diameter of the cell body) and the number of cells possessing at least one neurite. Cells were chosen randomly. The images were captured at 400x magnification, and the neurites were measured using Image J software (NIH, USA).

### 2.5. Viability Assays

5 × 10^3^ cells were plated in each well of a 96-well plate. For WST-1 assay, plates were incubated with WST-1 solution in a 1 : 10 ratio for 4 h at 37°C. The absorbance was measured at 450 nm using a Victor X3 multiwell plate reader (PerkinElmer). The IC_50_ value was determined by nonlinear regression analysis in Prism 8.0 software (GraphPad Software, San Diego, CA). When indicated, cells were pretreated for 4 h with 5 *μ*M BAPTA A/M added 48 h following PCN67 administration and the viability was assessed on the next day. Membrane permeability was determined in the presence of 7.5 *μ*M propidium iodide. Ru360 or cyclosporine A (CsA) was added 30 min before PCN67 treatment, and the viability was measured after 72 h. For experiments with 0 mM extracellular Ca^2+^, following 48 h of incubation with PCN67, the growing medium was changed to Hank's balanced salt solution with or without calcium for 4 h and cells were imaged 24 h after using a Leica DM4000 microscope. For some experiments, cells were cultures in the presence of galactose (10 mM) in RPMI medium for 5 days following treatment with PCN67 for another 24 h.

### 2.6. Primary Hippocampal Neurons

Hippocampal cultures were prepared from Sprague Dawley rat embryonic day 18 embryos. Briefly, the rat hippocampal CA1-CA3 region was dissected in PBS medium with 10 mM D-glucose and digested with 0.05% trypsin-EDTA in PBS for 20 min at 37°C. The dissociated tissues were centrifuged at 200*g* for 2 min and then triturated in the presence of DNase (100 U/ml) with a fire-polished glass pipet in Hank's balanced salt solution (HBSS) containing calcium and magnesium. Dissociated neurons were plated on nitric acid-treated 25 mm cover glass coated with poly-L-lysine in a plating medium. Four hours after plating, the medium was replaced with Neurobasal medium supplemented with 2% B27, 1 mM sodium pyruvate, and 2 mM L-glutamine. After four days, 4 *μ*M cytosine arabinoside was added to inhibit glial proliferation, and the neurons were transfected with the pEGFP-N3 vector using Lipofectamine LTX.

### 2.7. Axon Outgrowth Assay

The primary hippocampal neurons were grown until they reached either DIV4 or DIV14, and they were next treated with indicated PCN67 concentration. These stages were chosen because they correspond to distinct neuronal developmental processes such as axon formation and dendrite outgrowth in immature neurons (DIV4) or synaptogenesis associated with maturation (DIV14) as described previously [53]. 40 mM KCl was added to some cultures together with PCN67 as indicated. After two days, the neurons were imaged with Zeiss inverted scope and the photographs were processed using Corel Draw 11. The length of the longest neuron for approximately 20 neurons average per condition was measured for each experiment with Image J Simple Neurite Tracker plugin (NIH, USA).

### 2.8. Total RNA Isolation and Real-Time PCR

Total cellular RNA was isolated from PC12 cells using Trizol reagent based on the protocol provided by the manufacturer. Single-stranded cDNA was synthesized using M-MLV reverse transcriptase and oligo(dT) primers using 1 *μ*g of RNA. The gene expression was quantified using Maxima SYBR Green Master Mix in the conditions: 15 min at 95°C followed by 40 cycles at 95°C for 15 s, 60°C for 30 s, and 72°C for 30 s using the Abi Prism 7000 sequence detection system (Applied Biosciences). The specificity of primers was checked by running a melting curve. Each time, the expression level of the gene of interest was normalized to the endogenous expression of *Gapdh* and the relative fold change was calculated using the 2^-*ΔΔ*Ct^ method [54]. Either the primers were designed using the GenScript Primer Design Tool (USA) or their sequence was previously published elsewhere, as specified in Table 1.

### 2.9. Visualization of Necrotic Death with Flow Cytometry

1 × 10^6^ PC12 cells were double stained with Annexin V (to visualize apoptotic cells) and propidium iodide (sensitive to necrotic cells) for 15 min at 25°C in the dark using the Annexin V-FITC Apoptosis Detection Kit based on the information provided by the manufacturer and analyzed using the FACScan Becton Dickinson flow cytometer. The fluorescence was collected from 10^4^ cells, and the data were plotted with CellQuest Becton Dickinson software.

### 2.10. Lactate Dehydrogenase Release Assay

Lactate dehydrogenase (LDH) release from PC12 cells after drug treatment was measured with the LDH Cytotoxicity Assay Kit per manufacturer's protocol.

### 2.11. Cell Fractionation and Western Blot

PC12 cells were lysed on ice with RIPA buffer supplemented with 1 mM PMSF, 2 mM Na_3_VO_4_, and protein inhibitor cocktail for 30 min. The lysates were next centrifuged at 800 x *g* for 15 min at 4°C; the supernatant was boiled for 5 min in the Laemmli buffer and stored at -80°C for further use. Protein concentration was measured using Bradford reagent according to the manufacturer's instruction. Postnuclear fraction referred here as cytosolic was obtained based on the method of Blobel and Potter [57]. The purity was checked by using glyceraldehyde3-phosphate dehydrogenase (GAPDH) as a marker of cytosolic fraction and histone H3 for nuclear fraction. For Western blot, approximately 40 *μ*g of cytosolic proteins were separated using 10% SDS-PAGE and transferred to a nitrocellulose membrane with a semidry method. Membranes were blocked with 5% bovine serum albumin in TBS-T buffer (10 mM Tris-HCl, pH 7.4, 150 mM NaCl, and 0.05% Tween-20) for 1 h at room temperature and then incubated overnight at 4°C with primary antibodies: anti-HMGB1 (1 : 1000), anti-GAPDH (1 : 2500), or antihistone H3 (1 : 1000), followed by 4 h incubation with secondary antibodies (1 : 10000) coupled with alkaline phosphatase. BCIP/NBT, an artificial alkaline phosphatase substrate, was used to visualize immunoreactive bands. Membranes were densitometrically quantified using Image J software (NIH, USA). The results are presented as arbitrary units obtained after normalization of the marker protein.

### 2.12. Cellular ATP Content Measurement

ATP concentration in PC12 cells was determined using adenosine 5′-triphosphate (ATP) Bioluminescent Assay Kit on GloMax 20/20 luminometer and normalized to the protein content. For each set of measurements, a second negative control (no cells) was included, and background fluorescence was further subtracted from all other values. The results are presented as nmoles ATP/mg protein.

### 2.13. Live Cell Imaging

Simultaneous Ca^2+^ and membrane permeability live cell images were acquired using an inverted Zeiss Axio Observer 7 Marianas™ Microscope equipped with a X-Cite 120LED Boost White Light LED System and a high-resolution Prime™ Scientific CMOS digital camera that is controlled by a workstation loaded with SlideBook imaging and microscope control software (Intelligent Imaging Innovations, USA). Differentiated PC12 cells were transfected with a GcAMP3 calcium sensor using Lipofectamine LTX and treated with PCN67 as described in *Drug Treatment* subsection in the presence of 7.5 *μ*M propidium iodide. The images were acquired at 63x with the excitation time of 100 ms and one-hour interval time using the following filter set: GFP Exciter FF01-474/27, GFP Emitter FF01-525/45, and for propidium iodide: Exciter FF01-578/21 and Emitter FF02-641/75. The environmental control system (Okolab, USA) was used to keep constant experimental conditions (37°C, 5% CO_2_). The focus was automatically controlled by Definite Focus.2 (Zeiss, Germany). Regions of interest (ROIs) after background subtraction were normalized as Δ*F*/*F*_0_ and processed using Microsoft Excel software. ROIs were averaged over each condition. At least 10 cells were imaged per experimental condition, and all experiments were performed in triplicate.

### 2.14. Measurement of Mitochondrial and Plasma Membrane Potential

Mitochondrial membrane potential (*ΔΨ*_m_) was measured with TMRE (tetra-methyl-rhodamine-ethyl ester) whereas plasma membrane potential (*ΔΨ*_p_) was measured with DiSBAC_2_ (Bis-(1,3-diethylthiobarbituric acid) trimethine oxonol). Following 72 h of incubation with PCN67, PC12 cells were loaded with 25 nM TMRE or 1 *μ*M DiSBAC_2_ for 30 min at 25°C in the dark in a buffer containing 20 mM HEPES, pH 7.4, 2 mM CaCl_2_, 150 mM NaCl, 5 mM KCl, 1 mM MgCl_2_, and 10 mM glucose and the fluorescence of 10^4^ cells was recorded by a FACScan Becton Dickinson flow cytometer. The accompanying software analyzed the data. Cells incubated with 0.1% DMSO, used as a solvent for TMRE and PCN67, were monitored to record background fluorescence, which was later subtracted from the recordings.

### 2.15. Detection of ROS Level

The ROS level was measured with DCFH-DA (2′,7′-dichlorofluorescin diacetate) following 72 h treatment with PCN67. Briefly, at the end of the treatment, PC12 cells were washed with PBS and incubated with DCFH-DA at a final concentration of 10 *μ*M for 30 min at 37°C in the dark. After three washes with PBS to remove the excess of the dye, the fluorescence intensity was measured with a flow cytometer (Becton Dickinson) with 488 nm excitation wavelength and emission at 525 nm. During each experiment, 10^4^ cells were recorded. The background fluorescence was obtained with 0.1% DMSO.

### 2.16. Quantification of Lipid Peroxidation

The level of thiobarbituric acid reactive substances (TBARS) in PC12 cells was determined according to the method described previously [58]. The results were normalized to the protein content and are expressed as OD/mg.

### 2.17. Nitric Oxide Assay

Nitric oxide generation in PC12 cells following 72 h of PCN67 treatment was determined in phenol red-free media according to the method described previously [59]. Briefly, at the end of 72 h treatment, 100 *μ*l of culturing medium was transferred into a 96-well plate and mixed in a 1 : 1 ratio with Griess reagent (1% sulfanilamide/0.1% NED in 5% phosphoric acid) followed by 10 min incubation in the dark. The absorbance at 540 nm was measured using a Victor X3 multiwell plate reader (PerkinElmer). DMSO-treated cells were used as control.

### 2.18. Statistical Analysis

All data are expressed as mean ± S.E.M from at least 3 experiments unless otherwise stated. One-way or two-way ANOVA was performed with matching as appropriate. *P* values for experiments involving multiple comparisons were obtained by the Tukey post hoc testing, albeit *P* values for not all comparisons are indicated on the graphs. *P* < 0.05 was considered statistically significant. ^∗^*P* < 0.05, ^∗∗^*P* < 0.01, and ^∗∗∗^*P* < 0.001.

## 3. Results

### 3.1. The Dose-Dependent Effect of PCN67 on Differentiated PC12 Cell Survival

**Figure 1:**
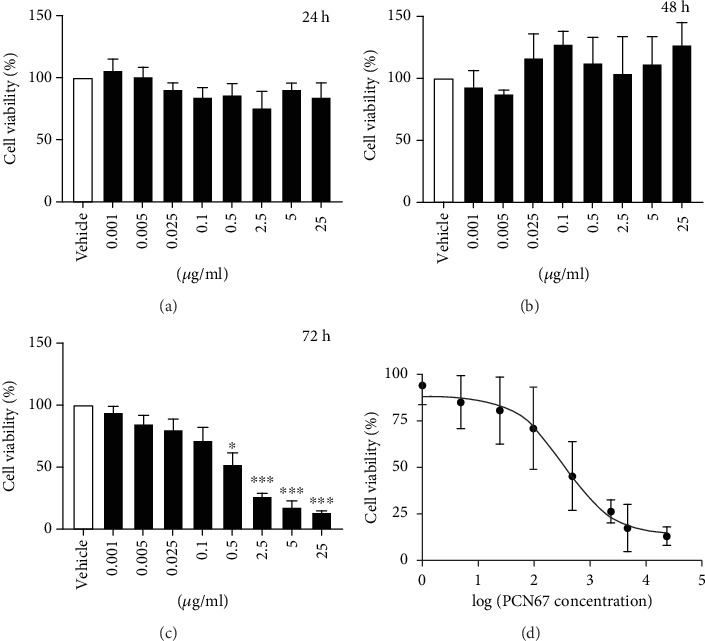
Dose- and time-dependent effect of PCN67 on differentiated PC12 cells. The cells were treated with PCN67 concentration ranging from 0.001 *μ*g/ml to 25 *μ*g/ml for 24 h (a), 48 h (b), and 72 h (c). The half-maximal inhibitory concentration (IC_50_) was determined based on viability data obtained following 72 h treatment using nonlinear regression analysis (d). Cell viability in vehicle-treated cells was taken as 100%. ^∗^*P* < 0.05 and ^∗∗∗^*P* < 0.001.

To assess *in vitro* neurotoxicity of PCN67, differentiated PC12 cells were exposed to a concentration ranging from 0.001 *μ*g/ml to 25 *μ*g/ml (Figures 1(a)–1(c)). A significant rise in cell mortality was visible after 72 h of incubation. The lowest tested concentration that caused a reduction in the number of viable cells was 0.5 *μ*g/ml (*F*(3,24), *P* < 0.05). Increasing PCN67 concentration only potentiated the prodeath effect. Based on the dose-response curve presented in Figure 1(d), the calculated IC_50_ value was 0.35 *μ*g/ml while the IC_10_ was 0.039 *μ*g/ml. In this context, we decided to employ the IC_50_ concentration and the highest concentration that did not produce significant cell death (0.1 *μ*g/ml) for the following experiments done 72 h after PCN67 treatment.

### 3.2. PCN67 Interferes with the Differentiation of PC12 Cells

**Figure 2:**
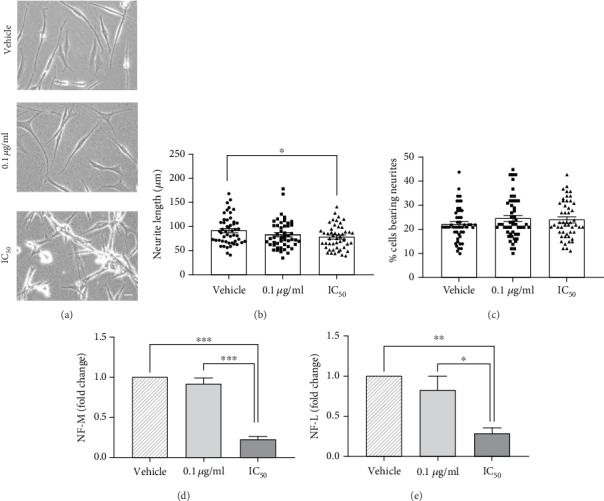
The effect of PCN67 on PC12 cell differentiation. (a) Morphology of control and PCN67-treated differentiated PC12 cells photographed under an inverted phase microscope with a CCD camera. Scale bar 10 *μ*m. (b) Quantification of an average length of neurites. The cell protrusion was counted as neurite when its length was at least twice of cell diameter. (c) Quantification of an average number of neurite-forming cells. Cells with at least one visible neurite were considered. (d) The expression of neurofilament M (NF-M) and (e) neurofilament L (NF-L) was assessed by real-time PCR 72 h after PCN67 treatment. The expression level in vehicle-treated cells was taken as 1. ^∗^*P* < 0.05, ^∗∗^*P* < 0.01, and ^∗∗∗^*P* < 0.001.

PC12 cells exposed to 1 mM dibutyryl-cAMP showed de novo outgrowth of bi- or tripolar neurites, which appeared mostly straight, occasionally forming branches in some areas. The body of the cell was mostly polygonal. We scored two parameters: the length of the neurites and the percentage of the cell bearing neurites, to evaluate the effect of PCN67 on the differentiation potential of PC12 cells. As shown in Figure 2(a), treatment with 0.1 *μ*g/ml of PCN67 for 72 h did not affect the general morphology, in contrast to the IC_50_ concentration, which led to some cell detachment, floating and losing the characteristic neuronal-like shape. Moreover, treatment with this concentration resulted in the formation of neurites shorter by 15% (77.1 *μ*m vs. 90.7 *μ*m in control, *F*(2,145), *P* < 0.05) but did not affect the number of cells bearing neuronal protrusions (Figures 2(b) and 2(c)). We subsequently analyzed the expression of neurofilament M (NF-M) and neurofilament L (NF-L) which are major components of the neuronal cytoskeleton and are frequently used as differentiation markers. No emphasis was given to neurofilament H, as it is expressed later during PC12 and sympathetic neuron differentiation, and its role in the early stages of this process is not defined [60]. Real-time PCR analysis showed a 77% reduction of the NF-M mRNA level (*F*(2,6), *P* < 0.001) after treatment with IC_50_ concentration of PCN67 when compared to the vehicle-treated control (Figure 2(d)). The expression level of NF-L measured at the same time point (Figure 2(e)) was reduced by 72% (*F*(2,6), *P* < 0.01). These results demonstrate that PCN67 in a dose-dependent manner may interfere with the gene expression of NF-L and NF-M associated with the differentiation of PC12 cells into neuronal phenotype.

### 3.3. Susceptibility of Primary Neurons to PCN67 Depends on the Maturation Stage

**Figure 3:**
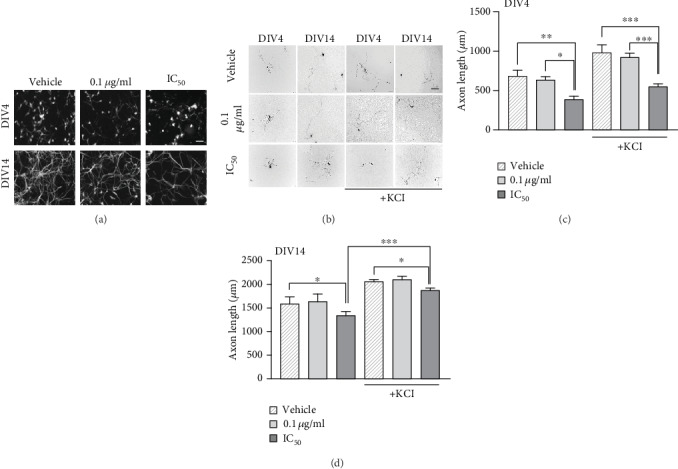
Toxicity of PCN67 depends on the maturation stage of primary hippocampal neurons. (a) The grayscale images of neuronal network photographed using an inverted phase microscope with a CCD camera. Scale bar 10 *μ*m. (b) Neurons transfected with GFP plasmid cultured in defined media in the presence or absence of KCl (40 mM). KCl was added together with PCN67, and the images were taken 2 days later. (c) Quantification of axon length at DIV4 and (d) at DIV14. The longest neurite was measured. ^∗^*P* < 0.05, ^∗∗^*P* < 0.01, and ^∗∗∗^*P* < 0.001.

Because differentiated PC12 cells acquire and retain several core features of primary neurons, we next checked whether the PCN67 effect observed in PC12 cells could be reproduced in hippocampal neurons *in vitro*. To search for any developmental-dependent effect, we used neurons at DIV4 and DIV14 that were susceptible to chronic KCl stimulation. It has been shown that KCl protects neurons from death resulting from overinhibition and from neurotrophic factor deprivation [61, 62]. Morphological observation showed significant disintegration on the neuronal network of DIV4 neurons treated with PCN67 at the IC_50_ concentration (Figure 3(a)). Moreover, measurement of the longest neurite at DIV4 showed a 43% reduction of the baseline length (*F*(5,94), *P* < 0.01) and no prosurvival effect of KCl (Figures 3(b) and 3(c)). Such effect was not observed for 0.1 *μ*g/ml PCN67 suggesting a dose-dependent action. The reduction of neurite outgrowth by 20% (*F*(5,102), *P* < 0.05) upon treatment with IC_50_ concentration of PCN67 was also demonstrated at DIV14; however, KCl was able to abolish the prodeath effect of PCN67 and rescue the axonal length (Figure 3(d)).

### 3.4. Induction of Necrosis by PCN67 Is Dose-Dependent

**Figure 4:**
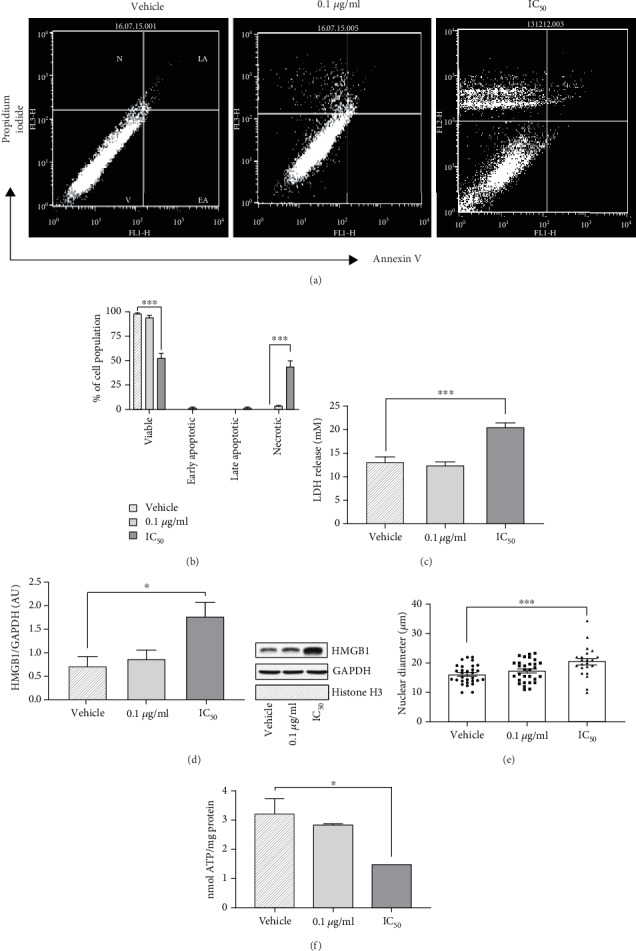
PCN67 treatment induces characteristics of necrotic death in differentiated PC12 cells. (a) Representative dot plots of necrotic/apoptotic cell distribution following 72 h of PCN67 treatment. Cells were stained with Annexin V and propidium iodide and analyzed with flow cytometry. (b) Quantification of viable (V), early apoptotic (EA), late apoptotic (LA), and necrotic (N) cells in a population. (c) Determination of plasma membrane integrity measured by the release of lactate dehydrogenase (LDH) 72 h following PCN67 treatment. (d) Western blot-based quantification of HMGB1 protein in cytosolic fraction collected from 72 h-treated cells. The results are presented as arbitrary units following normalization to the GAPDH level used as a marker of a cytosolic fraction. Histone H3, a protein marker of a nuclear fraction, was used to determine the purity of fractionation. (e) Average measurement of the nuclear diameter of differentiated PC12 cells after 72 h of PCN67 treatment. (f) The changes in the ATP level following 72 h of PCN67 treatment. The results were normalized to the protein level and are expressed as nmoles/mg. ^∗^*P* < 0.05 and ^∗∗∗^*P* < 0.001.

To investigate the nature of cell death, we next quantified Annexin V/propidium iodide (Ax-V/PI) staining (Figure 4(a)). The percentage of necrotic cells (Ax-V^−^/PI^+^) in the total cell population was increased to 45% (*F*(2,9), *P* < 0.001) only in PC12 cells treated with PCN67 at the concentration of IC_50_ (Figure 4(b)). In addition, virtually no early apoptotic cells (Ax-V^+^/PI^−^) were detected and the population of late apoptotic cells (Ax-V^+^/PI^+^) was of minor importance (less than 3%). To study this further, we measured the necrosis-associated membrane changes by detecting the release of lactate dehydrogenase (LDH) [63]. After treatment with the concentration corresponding to IC_50_ (Figure 4(c)), LDH presence in the extracellular media was significantly increased (*F*(2,36), *P* < 0.001). In parallel, we observed the accumulation of HMGB1 protein (high-mobility group protein B1) in the cytosol (Figure 4(d)), which is normally sequestered in the nucleus but upon induction of necrosis, it is exported to the cytosol to induce an inflammatory response [64]. Another feature of necrosis is nuclear swelling. The nuclear diameter following PCN67 treatment was significantly increased (Figure 4(e)) compared to vehicle-treated control (*F*(2,79), *P* < 0.001). In contrast to apoptosis, which is an ATP-dependent process, necrosis is energy-independent but results in ATP depletion [65]. In this context, upon treatment with PCN67 at IC50, we detected a cellular ATP level decrease by 55% (*F*(2,6), *P* < 0.05) (Figure 4(f)). Interestingly, exposure to 0.1 *μ*g/ml of PCN67 did not induce any necrotic features, pointing to a dose-dependent effect. Apoptotic markers were subsequently evaluated to eliminate the possibility of apoptotic death. However, neither PARP cleavage nor caspase 3/7 activation was observed (not shown). Collectively, our data suggests that PCN67 dose equivalent to IC_50_ induces the necrotic death of differentiated PC12 cells.

### 3.5. Increased [Ca^2+^]_c_ Does Not Contribute to PCN67-Induced Cell Death

**Figure 5:**
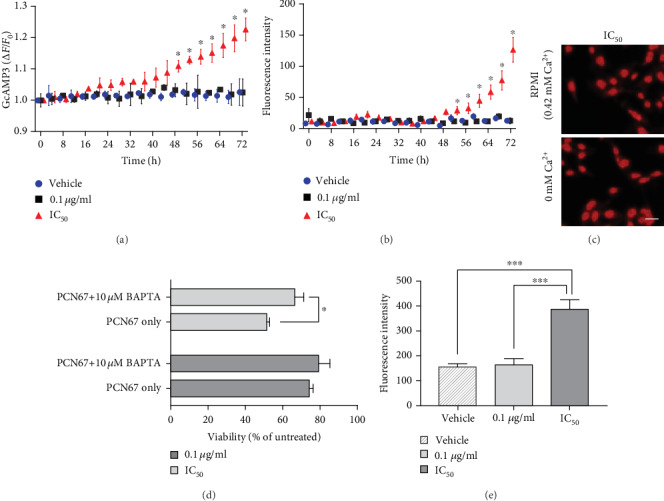
PCN67 induces intracellular calcium rise and plasma membrane depolarization. (a) Differentiated PC12 cells were treated with PCN67 for 72 h in the presence of intracellular calcium indicator—GcAMP3 and (b) propidium iodide (7.5 *μ*M). The cells were placed into an environmental chamber with controlled temperature and CO_2_ concentration, and the fluorescence changes were recorded every 4 h using an Axio Observer 7 Marianas™ Microscope equipped with 63x objective. The fluorescence of single cells was processed as *ΔF*/*F*_0_ after background subtraction. (c) Representative images of cells stained with propidium iodide (7.5 *μ*M) following treatment with PCN67 (IC_50_) for 72 h in the presence or absence of calcium in the culture media. Scale bar 10 *μ*m. (d) Chelation of intracellular calcium partially protected from cell death. BAPTA-AM (5 *μ*M) was added to the culture 48 h after PCN67 treatment, and the viability was determined the day after. Vehicle-treated cells were taken as 100%. (e) Plasma membrane potential was measured 72 h following PCN67 treatment using DiSBAC_2_ (1 *μ*M). Cells were loaded with a dye for 30 min, and the fluorescence was analyzed using flow cytometry. ^∗^*P* < 0.05 and ^∗∗∗^*P* < 0.001.

Another hallmark of necrosis is increased intracellular calcium concentration [65]. To verify whether the PCN67 mechanism of cell death involves disruption of calcium homeostasis, we used genetically encoded sensor GcAMP3 for a long-lasting recording of intracellular calcium changes. As shown in Figure 5(a), PCN67 applied at the concentration of IC_50_ but not 0.1 *μ*g/ml, resulted in a gradual increase in calcium level starting within the first 24 h and reaching significance at approximately 48 h and onward. After 2 days of treatment, a significant increase in propidium iodide uptake was visible, indicating plasma membrane hyperpermeability (Figure 5(b)). Further accumulation of propidium iodide was almost parallel to increasing GcAMP3 fluorescence. Next, we checked the requirement of extracellular calcium for PCN67-induced necrotic death. Replacing the regular calcium-containing growth media with Hank's balanced salt solution (calcium-free media) did not protect from compromised membrane permeability (Figure 5(c)), suggesting that extracellular calcium does not play a significant role. Pretreatment with intracellular calcium chelator—BAPTA A/M—after 48 h of PCN67 incubation, when a significant increase in calcium and membrane permeability started to be detectable, significantly, but only slightly preserved cell viability (Figure 5(d)). However, this effect was observed only at a higher dose of PCN67. These data illustrate that, although time-dependent calcium rise is noticeable after continuous treatment with IC_50_ concentration of PCN67, it does not have a noticeable effect on PCN67-induced necrotic death.

### 3.6. PCN67 Induces Necrosis-Associated Plasma Membrane Depolarization

Changes in plasma membrane potential can be monitored by using fluorescent dye—DiBAC4(3). Its uptake to the cell is driven by electrochemical gradient which results in increased fluorescence [66]. The measurements following 72 h of exposure showed elevated DiBAC4(3) fluorescence in cells treated with PCN67 concertation corresponding to IC_50_ value (2.5 times higher than that of control, *F*(2,9), *P* < 0.001) indicating plasma membrane depolarization (Figure 5(e)). This correlates closely with the increased membrane permeability to propidium iodide (Figure 5(b), 72 h). Lower doses of PCN67 did not provoke depolarization events or increased propidium iodide uptake. These data indicate that PCN67 may perturb the plasma membrane in a dose-dependent manner leading to membrane hyperpermeability, calcium overload, and necrotic death.

### 3.7. PCN67 Promotes Mitochondrial Dysfunction

**Figure 6:**
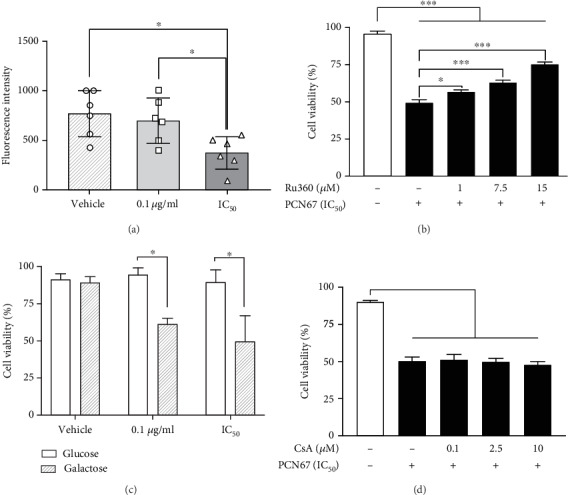
The role of mitochondria in PCN67-induced toxicity. (a) For measuring mitochondrial membrane potential, differentiated PC12 cells were cultured in the presence of PCN67 for 72 h and then loaded with TMRE (25 nM) for 30 min. TMRE-loaded cells were analyzed for fluorescence intensity using flow cytometry. Individual data points are shown. (b) Partial prevention of PCN67-induced death by Ru360. Ru360 was added 30 min before PCN67 treatment, and the viability was measured 72 h after. (c) Viability assessment of cells cultured in the presence of glucose (25 mM) or galactose (10 mM) supplemented RPMI for 5 days and then treated with PCN67 for 24 h. (d) PCN67-induced cell death is not inhibited by cyclosporine A (CsA). Differentiated PC12 cells were cotreated with PCN67 and CsA, and the viability was assessed 72 h later. ^∗^*P* < 0.05 and ^∗∗∗^*P* < 0.001.

To evaluate the contribution of mitochondria to cell death, the fluctuations in mitochondrial membrane potential (*ΔΨ*_m_) were monitored using TMRE (tetra-methyl-rhodamine-ethyl ester), which accumulates in the mitochondrial matrix according to the Nernst equation [67]. Treatment with IC_50_ concentration resulted in massive mitochondrial depolarization after 72 h (*F*(2,15), *P* < 0.05) which was reflected by a decrease in TMRE fluorescence (Figure 6(a)). To further elucidate the role of mitochondria in PCN67-induced cell death, an inhibitor of mitochondrial calcium uniporter—Ru360—was used. Pretreatment with Ru360 increased cell survival in a concentration-dependent manner (*F*(4,20), *P* < 0.05) when measured 72 h after exposure to PCN67 (Figure 6(b)). The presence of galactose in the medium, which is expected to upregulate mitochondrial oxidative phosphorylation and produce drug-induced mitochondrial failure [68, 69], sensitized cells to PCN67 (Figure 6(c)) as indicated by reduced viability detected already after 24 h. Interestingly, the same effect was also observed at a dose of 0.1 *μ*g/ml (*F*(1,24), *P* < 0.05). We next tested whether observed necrotic death involved mitochondrial permeability transition pore (mPTP) opening. Pretreatment with cyclosporin A, a potent mPTP inhibitor, failed to protect from PCN67-induced cell death (Figure 6(d)). These results collectively suggest that mitochondrial calcium may drive the PCN67-mediated necrotic process, which is however independent from mPTP.

### 3.8. The Role of Cellular Stress in HxCN-Induced Cell Death

**Figure 7:**
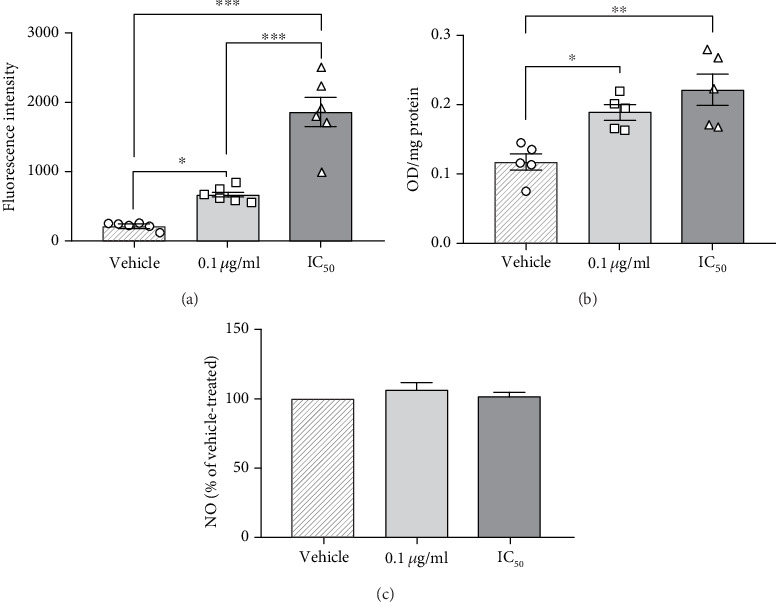
The effect of PCN67 on oxidative and nitrative stress. (a) Differentiated PC12 cells were treated with vehicle and PCN67 for 72 h. ROS generation was assessed with DCFH-DA (10 *μ*M) using the excitation and emission filters set at 488 nm and 525 nm. Individual data points are shown. (b) The oxidative damage of lipids by reactive oxygen species was assessed by measuring thiobarbituric acid reactive substances (TBARS), and the results are normalized to the protein level. Individual data points are shown. (c) The level of NO was determined using Griess reagent, and the absorbance readings at 540 nm were measured by a microplate reader. ^∗^*P* < 0.05, ^∗∗^*P* < 0.01, and ^∗∗∗^*P* < 0.001.

Based on the above results, we speculated that an increased ROS level could underlie compromised cell viability due to its interaction with mitochondria. Therefore, following 72 h of incubation with PCN67, cells were stained with DCFH-DA (2′,7′-dichlorofluorescin diacetate) to measure ROS. Significantly elevated ROS level (*F*(2,15), *P* < 0.001) was detected at IC_50_ concertation (Figure 7(a)). This corresponds to compromised membrane integrity measured by the release of LDH to the media (compare with Figure 5(c)). Interestingly, the average increase in ROS by (±SEM) 309 ± 10% was also noticeable at the lower concentration used when compared to vehicle-treated cells (100%). Because ROS can react with the polyunsaturated lipids, we next measured malondialdehyde levels to quantify membrane lipid peroxidation using TBARS assay. Exposure of PC12 cells to PCN67 resulted in marked increases in TBARS fluorescence for both concentrations (Figure 7(b)).

In order to dissect whether reactive nitrogen species could also contribute to observed necrotic death, we treated cells with PCN67 for 72 h and assessed the formation of a nitrile as a stable product of NO reaction with air using Greiss reagent. During the treatment, the cells were not challenged with lipopolysaccharide or *γ* interferon as differentiated PC12 cells have nNOS [70, 71]. Here, our aim was to assess if PCN67 is able to induce excessive NO generation. The data clearly indicate that neither PCN67 concentration stimulated or inhibited NO generation in relation to the vehicle-treated cells (Figure 7(c)).

## 4. Discussion

Neurons are a highly specialized type of cells required for cell-to-cell communication through axons and dendrites and information processing in the brain and spinal cord of the central nervous system. Under *in vitro* conditions, PC12 cells are a widely used model system to study neuronal processes, such as sprouting, excitability, and neurotransmitter release [72–74]. The PC12 line was originally derived from pheochromocytoma [73], a tumor arising from chromaffin cells of the adrenal medulla. Because chromaffin cells and sympathetic neurons originate from the neural crest, differentiated PC12 cells acquire both neuronal phenotype and several key features of functional neurons. These cells have also been employed to determine neuronal toxicity of a wide variety of environmental pollutants [75–78].

Polychlorinated naphthalenes, a group of substances widely used up to the 1980s, are still considered as a serious environmental threat as they have been found in practically all ecosystems, and their accumulation has also been confirmed in humans [22]. Despite some reports suggesting an interaction of these compounds with the neuronal physiology [45, 46], the underlying mechanisms and potential neurotoxic effects have not been studied so far. Therefore, in order to establish the model for PCN67 neurotoxicity and calculate the IC_50_, we performed a dose-dependent analysis (concentration ranging from 1 ng/ml to 25 *μ*g/ml) based on viability assay. The IC_50_ (0.35 *μ*g/ml) was additionally used to evaluate the nature of cell death. We employed the lowest PCN67 concentration (0.1 *μ*g/ml) that did not alter the viability of PC12 cells as compared to the control. Surprisingly, we found that already low PCN67 concentration of 0.1 *μ*g/ml which did not alter the viability of PC12 cells as compared to control induced cellular stress. Previous studies have shown that higher chlorinated polychlorinated naphthalenes, including PCN67, did not affect the viability of MCF-7 cells, when treated with the concentration of 100-10.000 pg/ml for 72 h [79]. However, at 1000 pg/ml, they increased caspase-8 activity and PCN67 additionally stimulated caspase-9, suggesting that the early molecular stages of death cascades may be initiated at much lower doses. Furthermore, it was demonstrated that the mixture of polychlorinated naphthalenes commercially available as Halowax 1051 downregulated aryl hydrocarbon receptor (AhR) when applied at 1 ng/ml and 10 ng/ml [80]. The disruption in AhR signaling has been shown to impair neuronal growth and development in *C. elegans* [81], affect arborization of sensory neurons in *Drosophila* [82], and diminish neuronal differentiation in dentate gyrus of the hippocampus [83]. Polychlorinated naphthalenes are potent agonists of AhR [84]; therefore, we cannot exclude that the effect of PCN67 on *in vitro* differentiation observed in our study can be, at least in part, mediated by AhR signaling. We demonstrated here that PCN67 treatment at IC_50_ concentration significantly decreased the number of neurite-forming cells and the average length of neurite in PC12 cells. A similar effect on axon length was also observed in primary hippocampal neurons, although more advanced culture stages (DIV14) seemed to be more resistant to PCN67 toxicity than the younger one (DIV4). Although no *in vitro* studies explored the effect of PCN67 on primary neurons so far, the finding of developmental-dependent sensitivity appears to be in line with our earlier work, showing the fetotoxic and embryotoxic effect of PCN67 at the doses that did not cause maternal toxicity [33]. Moreover, our results are supported by earlier reports, showing the effect of 2,3,7,8-tetrachlorodibenzo-*p*-dioxin (TCDD) on viability and neuronal outgrowth in different *in vitro* models [85–87]. However, some studies also showed enhanced binding of nerve growth factor to its receptor in PC12 cells in the presence of PCBs [88]. Thus, the neurotoxic effect of PCNs, which may be expected in analogy to TCDD and PCBs, would be determined by the biochemical and neurochemical properties of individual congeners.

One of the main characteristics of PCNs is high lipophilicity which allows to anticipate high penetration through biological membranes and rapid modification of membrane homeostasis and permeability. In our study, the earliest molecular change observed following PCN67 treatment at the IC_50_ concertation was mitochondrial membrane depolarization and significant depletion of ATP. In parallel to increased permeability of the plasma membrane to small molecules (e.g., propidium iodide), that started to be detectable 2 days following the treatment, we observed a gradual increase in cytosolic calcium concertation. Calcium is immediately taken up by mitochondrial calcium uniporter (MCU) which has been linked to H_2_O_2_ production, electron transport chain alteration, and disruption of *ΔΨ*_m_ homeostasis [89]. A significant drop in the ATP level and thus the increase in AMP to ATP ratio observed for instance in Caco-2 cells treated with environmental pollutant TCDD [90], may activate AMP kinase that regulates mitochondrial homeostasis through the PGC1*α* pathway. AMP kinase-PGC1*α*-dependent control of mitochondrial metabolism is expected to limit ROS production [91], which in concert with mitochondrial calcium overload could promote mPTP-driven cell death. Although we excluded the contribution of mPTP, our findings that (1) PCN67 is more effective in glucose-free media and (2) inhibition of MCU partially reversed the neurotoxic effect of PCN67, suggest a key functional role of mitochondria in PCN67-induced necrotic death.

Compounds that target mitochondrial metabolism usually induce cellular stress [92, 93]. Since PCN67 interfere with mitochondria and affect energy metabolism, its neurotoxicity could be associated with the production of free radical species. A dose-dependent elevation in ROS production revealed in this study not only confirmed PCN67 action on mitochondria but also suggested its detrimental effect on plasma membrane integrity as determined by increased lipid peroxidation and permeability to LDH. Generation of ROS and associated peroxidation of lipids were reported earlier in rat hepatocytes following PCN67 [33, 40] and PCN mixture treatment [43, 44] or neuronal cells exposed TCDD and PCBs [94, 95] suggesting a common mechanism. Interestingly, the cellular effects of oxidative stress were also detected for PCN67 concentration of 0.1 *μ*g/ml, which did not affect *ΔΨ*_m_ and plasma membrane permeability. We hypothesize that the observed dose-dependent effect can be attributed to the potency of PCN67 to influence cellular antioxidant defense, in particular GSH/GSSG redox potential. Indeed, it has been demonstrated that prolonged administration of PCNs *in vivo* decreased GSH concentration [43]. Therefore, lack of morphological and biochemical symptoms of cell death in the presence of increased ROS level at the concentration of 0.1 *μ*g/ml may imply that cellular defense systems are still sufficiently enough, to counteract oxidative damage. Moreover, the lack of damage to neurites could suggest that neuronal viability and function are preserved during short time exposure (72 h in this study) to lower PCN67 concentrations. However, since PCNs are highly bioaccumulative, it is plausible that exposure to even lower doses during a long-time period and concomitant gradual accumulation may ultimately lead to neurophysiological dysfunction. To support that, a variety of neurobehavioral tests performed on rats exposed to PCN67 showed dose-dependent impaired long-term memory, reduced pain threshold, and stress-induced analgesia [45]. However, the molecular targets for PCN67 in the brain have not been revealed so far.

Neuronal injury may activate other nonneuronal cells in the CNS-like astrocytes and microglia to trigger a neuroinflammatory response. Therefore, we investigated whether PCN67-induced damage and necrotic death could also involve excessive production of NO, one of the main mediators of inflammation. We found no significant changes between vehicle- and PCN67-treated groups. Although no studies demonstrated the effect of PCNs on NO production, data derived from persistent organic pollutants with a similar mechanism of action indicated no evidence of nitrative stress in cerebellar neurons and in rat hypothalamus [96, 97]. Since excessive production of NO and consecutive active inflammatory processes are usually associated with cytotoxicity, lack of enhanced NO production in response to PCN67 indicates that NO did not contribute to PCN67-induced neurotoxicity.

## 5. Conclusion

It has been documented that dysfunctional mitochondria or altered synaptic transmission due to changes in the neuronal network could be associated with several neurobehavioral disorders such as anorexia [98], depression [99], or memory loss [100]. The findings of neurite damage and impaired physiological function of mitochondria revealed in our study suggest that these phenomena may be one of the contributing factors for cognitive defects during long-term exposure to PCN67. On the other hand, since profoundly altered homeostatic control of energy balance and neuronal loss is frequently observed in schizophrenia [101] and Alzheimer's disease [102], the dose-dependent neurotoxicity and changes in the ATP level and lactate production could highlight a potential higher susceptibility for specific individuals exposed to PCNs to some neurodegenerative disorders. In this regard, further studies are vital to shed light on the possible implication of PCN in neurological disorders.

## Figures and Tables

**Table 1:** The list of primers used for gene expression quantification. The sequence for *Nefl* and *Gapdh* was taken from [55, 56], respectively. The primers for *Nefm* were designed as described in Section 2.8.

Gene name	Sequence 5′-3′	NCBI number
*Nefm* (*neurofilament M*, *NF-M*)	F: ATCACTTGGAGGAAGACATCCACCGG R: TTCCTCTGCAATGACTGTAGGGC	NM_017029

*Nefl* (*neurofilament L*, *NF-L*)	F: AGACATCAGCGCCATGCA R: TTCGTGCTTCGCAGCTCAT	NM_031783.1

*Gapdh*	F: GGTTACCAGGGCTGCCTTCT R: CTTCCCATTCTCAGCCTTGACT	NG_028301.1

## Data Availability

The raw data used to support the findings of this study have been deposited in the 4TU.ResearchData repository (DOI: 10.4121/uuid:5eb7226e-7546-4960-91bd-8b806c09668a).
